# Salmon lice – impact on wild salmonids and salmon aquaculture

**DOI:** 10.1111/jfd.12061

**Published:** 2013-01-13

**Authors:** O Torrissen, S Jones, F Asche, A Guttormsen, O T Skilbrei, F Nilsen, T E Horsberg, D Jackson

**Affiliations:** 1 Institute of Marine ResearchBergen, Norway; 2 Faculty of Biosciences and Aquaculture, University of NordlandBodø, Norway; 3 Pacific Biological Station, Fisheries and Oceans CanadaNanaimo, British Columbia, Canada; 4 Department of Industrial Economics, University of StavangerStavanger, Norway; 5 UMB School of Economics and Business, Norwegian University of Life SciencesÅs, Norway; 6 Department of Population Genetics and Ecology, Institute of Marine ResearchBergen, Norway; 7 Department of Biology, Sea Lice Research Centre, University of BergenBergen, Norway; 8 Department of Pharmacology and Toxicology, Norwegian School of Veterinary ScienceOslo, Norway; 9 Marine InstituteGalway, Ireland

**Keywords:** aquaculture, Atlantic salmon, *Lepeophtheirus salmonis*, management, Pacific salmon, socio-economic impact

## Abstract

Salmon lice, *Lepeophtheirus salmonis*, are naturally occurring parasites of salmon in sea water. Intensive salmon farming provides better conditions for parasite growth and transmission compared with natural conditions, creating problems for both the salmon farming industry and, under certain conditions, wild salmonids. Salmon lice originating from farms negatively impact wild stocks of salmonids, although the extent of the impact is a matter of debate. Estimates from Ireland and Norway indicate an odds ratio of 1.1:1-1.2:1 for sea lice treated Atlantic salmon smolt to survive sea migration compared to untreated smolts. This is considered to have a moderate population regulatory effect. The development of resistance against drugs most commonly used to treat salmon lice is a serious concern for both wild and farmed fish. Several large initiatives have been taken to encourage the development of new strategies, such as vaccines and novel drugs, for the treatment or removal of salmon lice from farmed fish. The newly sequenced salmon louse genome will be an important tool in this work. The use of cleaner fish has emerged as a robust method for controlling salmon lice, and aquaculture production of wrasse is important towards this aim. Salmon lice have large economic consequences for the salmon industry, both as direct costs for the prevention and treatment, but also indirectly through negative public opinion.

## Introduction

The Danish–Norwegian bishop, Erik L. Pontoppidan (1698–1764), was probably the first to describe the salmon louse in print by his description of ‘great schools of salmon moving from the sea into fresh water, partly to refresh themselves, and partly to rid themselves by rubbing and washing in the swift currents and waterfalls, of a kind of greenish vermin called ‘Laxe-Luus,’ attached between the fins, plaguing it in the heat of spring’ (Berland & Margolis 1983). Bishop Pontoppidan's report suggests that salmon lice were abundant on Atlantic salmon around 1750 in sufficient quantities to induce signs of discomfort or wounds and that ‘salmon louse’ was a commonly used name for the parasite. A report in 1940 from the Moser River (Nova Scotia, Canada) describes severe salmon louse infections and associated deaths: ‘fish, which were apparently freshly ascended from the estuary, carried hundreds of lice … some of the grilse had an almost complete layer of lice extending from the posterior edge of the eyes to the caudal peduncle on the dorsal part of the body with also a few lice around the anal and pelvic fins’ (White 1940). These accounts suggest a substantial annual variation in salmon louse infection rates. Salmon lice on Atlantic salmon caught in rivers were once considered a sign of prime quality as this indicated that the fish only recently entered the river and had not yet suffered the decline in quality associated with sexual maturation. The initial scientific interest in salmon lice was low, however, with the publication of only a few reports until salmon lice began to cause problems for the aquaculture production of Atlantic salmon (Brandal, Egidius & Romslo 1976; Brandal & Egidius 1977; Johannessen 1978).

**Figure 1:**
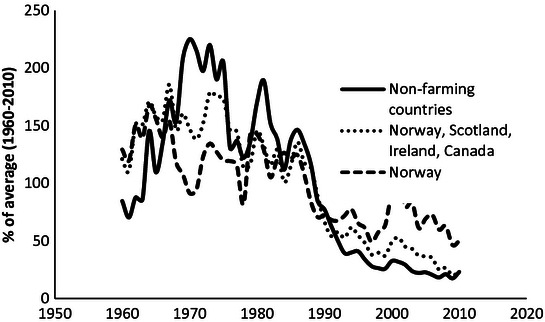
Relative nominal catch of Atlantic salmon from 1960 to 2010 in ‘non-farming countries’ (USA, Russia, Iceland, Sweden, Denmark, The Faeroe Islands, Greenland the UK – except Scotland, France and Spain), ‘Norway, Scotland, Ireland and Canada’, and ‘Norway’(NASCO 2011). The Faroe Islands is included among ‘non-farming’ as their salmon fishery is a marine fishery.

Aquaculture production of Atlantic salmon, *Salmo salar* L., reached approximately 1.5 million tons in 2009, with Norway being the largest producer, followed by Chile, the United Kingdom and Canada (Torrissen *et al*. 2011). Wild stocks of Atlantic salmon declined during the same period (Anon 2011; NASCO 2011), and the nominal catch in 2010 was 1589 tons or approximately 0.1% of the total landings of wild and cultured Atlantic salmon (Fig. 1). In general, sea pen culture of salmon has greatly increased our knowledge of marine pathogens (Bakke & Harris 1998). The salmon louse has been a serious problem for the Atlantic salmon farming industry since the 1970s (Brandal *et al*. 1976; Brandal & Egidius 1977), and the salmon louse has a greater economic impact than any other parasite (Costello *et al*. 2004). The year-round high density of hosts provides the ideal conditions for salmon lice. Not surprisingly, within a few years of the onset of intensive salmon aquaculture, salmon farms were proposed to be the primary sources of salmon louse epizootics on wild sea trout in Ireland (Tully & Whelan 1993).

The apparent inverse relationship between the Atlantic salmon aquaculture production and the catch or abundance of wild salmon has led to discussions and conflicts between the salmon farming industry and society, often represented by different non-governmental organizations (NGO). The core of the conflicts has been disagreement on the scale of the impact of salmon lice or their therapeutants on the decline of wild salmon populations or non-targeted species (http://www.worldwildlife.org; http://www.puresalmon.org; http://www.mangroveactionproject.org; http://www.farmedanddangerous.org). In this respect, there is a common failure to recognize that a correlation between the two sets of data does not necessarily indicate a cause–effect relationship (McVicar 2004). The current controversy arises partly from a lack of good data, leading to over-interpretation and possibly misinterpretation of the available information (McVicar 2004).

Aquaculture production of salmonids in open-cage systems will probably always be challenged by salmon lice and, as with many other diseases in farmed animals and humans, the management of salmon lice infestations will remain an ongoing battle. In this battle, the farming industry will pursue multiple strategies to control salmon lice infestation rates to acceptable levels and the parasite will demonstrate a capacity to adapt to these efforts. The 9th International Symposium on Sea Lice was held in Bergen Norway in May 2012. The intention of this article is to summarize the current knowledge of salmon louse biology, including the epidemiology, host interaction and impact on wild fish, as well as advances in the treatment, control and management of salmon lice. We also discuss salmon lice from a social and economic perspective.

## Socio-economic considerations

The main social impact of salmon is to create jobs and livelihood. Aquaculture is the largest activity in this respect, but the recreational and commercial wild fisheries are also substantial. Negative impacts on wild salmon stocks from farming activities are perceived to reduce wild catches for anglers and decrease the demand for fishing licences (Olaussen & Liu 2011). While fish farms may displace fishermen from their traditional harvesting grounds, farming may also lead to local increases in catches as wild fish forage on waste feed available near farms. Salmon aquaculture also provides an indirect livelihood for a number of people, such as suppliers, administrators and processors. Olavsen *et al*. (2011) estimate that the number of people employed in aquaculture-related jobs is twice that of people directly involved in the aquaculture core operation. The recent loss of more than 20 000 jobs in salmon farming and processing as a result of infectious salmon anaemia and salmon lice in Chile provides a dramatic example of the importance of disease and parasite management (Alvial *et al*. 2011).

The salmon lice issue receives attention partly because of the potential spread of lice from farmed to wild salmon and partly because of interactions between farms. It is necessary to distinguish between the costs to individual farms associated with salmon louse infestation and the social costs of salmon lice discharged from those farms. Costs to individual farms include those associated with lost production due to disease or fallowing and with treatment. Costs associated with salmon lice that impinge other farms or the broader ecosystem include higher production costs for other farms due to elevated infestations or reduced catches of wild salmon due to increased mortality of wild salmon stocks (Asche, Guttormsen & Tveterås 1999).

Farm-specific costs due to salmon lice are relatively well understood. A farm will normally treat against sea lice when production costs are increased due to a reduced growth rate, increased feed conversion rate and reduced marketability due to skin injuries at a levels exceeding the treatment costs. For the 2006 global production of Atlantic salmon (1.6 million tons), it is estimated that lice treatment cost the industry approximately 305 million € (Costello 2009).

The influence of salmon lice on production costs at one farm tends to influence several farms in a region (Tveterås 2002). For the industry in the region, this provides a justification for regulations to limit this negative externality.

Criticism of the salmon farming industry for how they have handled the salmon louse problem has influenced the design of regulations and licences to operate. For example in Norway, concerns with respect to salmon lice led to a postponed implementation and possibly abandonment of an increase in the maximum allowable biomass (MAB) (Asche & Bjørndal 2011). The strong negative publicity on the sea lice issue may also influence the public reputation of salmon and the salmon industry, reducing the demand and consequently the price for salmon. As there is a global market for salmon (Asche *et al*. 2005), however, this effect is most likely limited.

A thorough bioeconomic model that accounts for the externalities caused by neighbourhood farms and regulations is required to fully determine the cost of salmon lice. Limited knowledge is available regarding the level of this cost, but it is likely to vary substantially between farms and regions. For the economic sustainability of salmon farming, it is important that regulatory measures are evaluated with respect to the economic as well as environmental impact. In general, regulatory design and the compatibility of regulatory measures with fish farmers′ incentives will significantly influence costs incurred by the regulations as well as the effectiveness of the regulations. Regional bioeconomic models may best evaluate the economic impacts of different regulatory measures.

On the basis of production growth and employment, salmon aquaculture is a success story. Innovations that enhance productivity and improve competitiveness are the main factors behind the growth (Guttormsen 2002; Asche 2008). However, salmon aquaculture has the potential to cause negative externalities both by excessive use of resources in the surrounding ecosystem and through interactions with wild stocks as exemplified by salmon lice. Hence, regulations and good governance are necessary to establish a sustainable industry (Smith *et al*. 2010). Regulations, however, directly influence the competitiveness of an industry, and thus, regulatory design is very important for the economic and societal sustainability of the industry. For example, regulations have eroded the competitiveness of the salmon production industries in Canada and Scotland (Asche & Bjørndal 2011). Hence, if salmon aquaculture is to be sustainable, the lice challenge must be addressed with cost-efficient measures that allow firms and societies to continue to thrive with the industry.

## General biology

The salmon louse, *Lepeophtheirus salmonis*, has a circumpolar distribution in the Northern Hemisphere (Kabata 1979) and is principally a parasite of salmonids in the genera *Salmo*, *Oncorhynchus* and *Salvelinus*. Atlantic salmon post-smolts leave coastal waters quickly after migration from the rivers and are unlikely to represent a significant source of salmon louse larvae in coastal waters (Butler 2002). Prior to salmon aquaculture operations, the year-round presence of sea trout, *Salmo trutta* L., in coastal waters probably supported a local over-wintering population of salmon lice (Butler 2002; Rikardsen 2004). Returning Atlantic salmon are a significant source of lice with a nearly 100% prevalence, and the louse population comprises predominantly ovigerous females (Copley *et al*. 2005; Jackson *et al*. 2012), particularly in areas with few salmon farms.

The salmon louse is a stenohaline copepod whose survival and development are optimal in high-salinity sea water. In this context, it is informative to consider the life history of *L. salmonis* as a series of behavioural and reproductive strategies to cope with an obvious dilemma: survival in an environment that ranges from low host density and high salinity to one of high host density and ultimately fresh water. For populations of *L. salmonis* that occur on wild salmon, reproduction and transmission of the copepod are coordinated with the two intervals in the life history of the salmon in which host density and salinity are optimized: in coastal ecosystems during the spawning migration of adults and following the return of juvenile salmon to the ocean. Alternatively, on captive salmon populations that reside in net pens in high-salinity coastal environments for 12–24 months, the opportunities for reproduction and transmission are theoretically continuously high, with increased opportunity for more intense and therefore harmful infections and for shedding larvae at higher densities into the surrounding water column over prolonged periods. Much of the current scientific interest in *L. salmonis* results from a new host–parasite system in which wild salmonids interact with populations of captive salmon in these high-salinity coastal ecosystems.

The life cycle of *L. salmonis* comprises non-feeding planktonic larvae (nauplii), infective planktonic copepodites, immature chalimi embedded on the host skin and mobile pre-adults and adults that move freely over the host skin (Hayward, Andrews & Nowak 2011). The infectious larval copepodid subsists entirely on endogenous lipid reserves and therefore devotes its time entirely to host-finding and attachment via a suite of adaptive behavioural traits. These traits include positive phototaxis, positive semiotaxis and positive rheotaxis, which confer to the larval copepod the ability to display diurnal vertical migrations, respond to waterborne gradients of host-derived chemicals and move towards vibrations of host origin, respectively (Heuch, Parsons & Boxaspen 1995; Heuch & Karlsen 1997; Aarseth & Schram 1999; Bailey *et al*. 2006). In addition, attachment following settlement is mediated by chemoreceptors associated with the copepodid antennules (Bron, Sommerville & Rae 1993). At compatible temperatures and salinities, the free-swimming copepodid survives up to 7 days (Stucchi *et al*. 2011), although energy content and attachment to the host decline between 3 and 7 days (Tucker, Sommerville & Wootten 2000).

Although *L. salmonis* occurs throughout the Northern Hemisphere, genetically distinct variants occur in the Atlantic and Pacific Oceans. Microsatellite data from six loci revealed significant variations between populations from the Pacific Ocean and Atlantic Ocean (Todd *et al*. 2004). Similarly, Tjensvoll, Glover & Nylund (2006) reported differences in the mitochondrial genome between a population of *L. salmonis* from Japan and the Atlantic Ocean. Subsequently, based on samples obtained from nine populations throughout the Pacific Ocean, it was found that nuclear genes differ, on average, by 3.2% and the mitochondrial genome by 7.1% between Pacific and Atlantic forms of the parasite (Yazawa *et al*. 2008). This finding is consistent with the geographic isolation and divergence of salmon belonging to *Oncorhynchus* and *Salmo* 11–24 million years ago (McKay, Devlin & Smith 1996). A weak but statistically significant genetic differentiation was detected among salmon lice sampled in the North Atlantic, suggesting that salmon lice display a subtle population structure throughout this range. A positive relationship between geographic and genetic distance has also been reported (Glover *et al*. 2011). A related study failed to detect a population structure in *L. salmonis* from the Pacific Ocean (Messmer *et al*. 2011). Gene flow among populations in both oceans therefore appears high and most likely results from the association of the parasite with highly migratory hosts.

## Lice–host interactions

The host suffers significant physiological and pathological consequences due to its interactions with *L. salmonis* that are largely dependent on the number and developmental stage of the copepod. In Atlantic salmon, while physiological changes are evident during infection by the chalimus stages, the feeding behaviour of the pre-adult and adult copepods combined with their unrestricted mobility on the host is responsible for the most severe pathophysiological consequences (Finstad *et al*. 2000). Heavy infections lead to erosion of the epidermis with exposure of the dermis and, in severe cases, skeletal muscle. Morbidity and mortality resulting from infection with *L. salmonis* are rare among wild salmon (Johnson *et al*. 1996). Prior to the systematic application of efficacious treatments, severe infections were common among netpen-reared salmon (Johnson *et al*. 2004). More common, and therefore of greater significance, are the subclinical physiological consequences of infections, including stress, changes in blood glucose or electrolytes, reduced haematocrits and reduced swimming performance (Wagner, Fast & Johnson 2008). These effects are strongly influenced by the number of copepods and their stage of development. In contrast, the effects of *L. salmonis* vary among species of Pacific salmon (*Oncorhynchus* spp.) because of differences in natural susceptibility to the parasite. Salmon lice are rejected more rapidly by coho, *Oncorhynchus kisutch* (Walbaum), and pink, *O. gorbuscha* (Walbaum), salmon than by Chinook, *O. tshawytscha* (Walbaum), and chum, *O. keta* (Walbaum), salmon, and, in contrast to chum salmon, juvenile coho and pink salmon avoid the clinical consequences of infections (Johnson & Albright 1992; Jones *et al*. 2007). Pink salmon first enter the ocean at a mean weight of approximately 0.3 g and may be exposed to *L. salmonis* at this time, thus providing a unique opportunity to assess the ontogeny of innate resistance to *L. salmonis*. Mortality is significantly elevated among salmon weighing 0.3 g, but not among larger size classes following laboratory exposure, suggesting that the onset of resistance to *L. salmonis* occurs in pink salmon between 0.3 and 0.7 g (Jones, Kim & Bennett 2008a). Subsequent research confirmed this initial finding and, based on body ion and maximum swimming velocity tests, defined a ‘no-effect’ threshold of 0.5 g for *L. salmonis* infections in juvenile pink salmon (Nendick *et al*. 2011; Sackville *et al*. 2011). Further, these studies showed that in the smallest salmon, salmon lice induce changes to the skin that result in the loss of ionoregulatory homoeostasis.

A more thorough understanding of the mechanisms of innate and acquired defence responses of salmonids to *L. salmonis* may form the basis of novel management strategies. Differences in susceptibility to *L. salmonis* among salmon species were initially associated with histological evidence of local inflammatory processes at the infection site (Johnson & Albright 1992). Subsequent studies indicated a relationship between susceptibility and the reduced or delayed expression of a relatively small number of proinflammatory genes (Fast *et al*. 2006; Jones *et al*. 2007; Jones, Fast & Johnson 2008b). Coincidently, *L. salmonis* feeding behaviour consists not only of mechanical abrasion and consumption of host tissues, but also the production of parasite excretory/secretory products (SEPs) (Fast *et al*. 2003). The *L. salmonis* SEPs contain prostaglandin E_2_ (Fast *et al*. 2004), and *in vitro* studies showed that the SEPs trigger a significant dysregulation of immune-related genes in either primary or immortalized Atlantic salmon head kidney leucocytes (Fast, Ross & Johnson 2005; Fast *et al*. 2007). Further research on the differential immunomodulatory capacities of *L. salmonis* SEPs from Atlantic- and Pacific-type *L. salmonis* (Yazawa *et al*. 2008) is necessary to better define the resistance characteristics among salmon species.

Management of *L. salmonis* will also benefit from improved salmon breeding programmes and the application of genomic technologies. In Atlantic salmon, intraspecific heterogeneity in resistance to *L. salmonis* is observed among spawning stocks and full-sib families (Glover *et al*. 2004, 2005; Kolstad *et al*. 2005; Gjerde, Odegard & Thorland, 2011). Although the heritability of louse counts ranges from 0.07 to 0.33, indicating a genetic basis for differences among families, there is disagreement regarding the identity of acquired immunological markers of resistance to the parasite (Glover *et al*. 2007; Gharbi *et al*. 2009). An improved understanding of innate markers may be necessary to explain resistance to *L. salmonis* (Gharbi *et al*. 2009). Global gene expression studies have begun to elucidate the pathways of innate and acquired salmonid defence responses to *L. salmonis* infections (Skugor *et al*. 2008; Sutherland *et al*. 2011; Tadiso *et al*. 2011). There is currently very little evidence that Atlantic salmon mount a protective immune response to either *L. salmonis* infection or immunization with parasite antigens (Grayson *et al*. 1991; Reilly & Mulcahy 1993; Roper *et al*. 1995). Furthermore, Atlantic salmon remain susceptible to reinfection following recovery from *L. salmonis* (Raynard *et al*. 2002). Thus, the development of a vaccine against *L. salmonis* in Atlantic salmon remains a long-term goal and may depend on the selection of suitable salmon strains in which natural resistance is already high.

However, salmon lice will also be under the same evolutionary mechanisms as other animals. Intensive farming will alter the selection criteria such as life-history traits and virulence where frequent use of antiparasite drugs and increased host density may select for faster production of parasite transmission stages via earlier reproduction and increased early fecundity (Mennerat *et al*. 2010, 2012). They also show a clear link between early reproduction, increased fecundity and increased virulence.

## Salmon louse population dynamics

### Wild salmonids

Within Europe, significant anthropogenic influences have affected the historical native range of Atlantic salmon; in many rivers, salmon can only breed with human intervention. Many populations are exposed to pollution, changed temperature regimes and low or managed water flow. The effects of diseases and parasites on the salmon populations are particularly difficult to determine (Bakke & Harris 1998). Salmon lice are endemic on wild Atlantic salmon, sea trout and Arctic charr, *Salvelinus alpinus* (L.), in the North Atlantic. In Ireland, a 20-year monitoring programme indicates that the prevalence of *L. salmonis* is consistently in excess of 90% (Jackson & Minchin 1992; Costelloe *et al*. 1998; Copley *et al*. 2005; Jackson *et al*. 2012). The reported levels are consistent with data from Scotland (Todd *et al*. 2000) and Norway (Berland 1993). The near-shore population structure of lice infesting wild Atlantic salmon is quite different from that of populations observed in the offshore or returning adults. Samples of Atlantic salmon (*n* = 547) captured in an interceptory offshore drift net fishery over a 3-year period showed a prevalence approaching 100% for *L. salmonis,* and the population comprised largely of adult lice. Atlantic salmon captured in an inshore and estuarine draft net fishery (*n* = 381) over a 2-year period had a much higher proportion of juvenile lice and over 30% of samples contained fish with chalimus stages (Jackson *et al*. 2012, this issue). The presence of chalimus stages indicates recent successful infestation and points to re-infestation of returning adult wild populations in inshore waters with a potential for amplification of louse levels that are not evident in offshore stocks.

Salmon louse populations on wild Atlantic salmon show a mean abundance of 6–33 per fish (D. Jackson, unpubl. data, Copley *et al*. 2005; Costelloe *et al*. 1998; Jackson *et al*. 2012). Adult female mean abundance varies from 0 to 17 per fish. Female *L. salmonis* from wild salmon are larger than those recorded on farmed fish (Jackson & Minchin 1992; Copley *et al*. 2005) and have higher fecundity (Jackson & Minchin 1992). There is good evidence for a pulse of infestation pressure in the spring as water temperatures rise and the returning adult wild salmon arrive off the coast (Jackson *et al*. 1997). This spring pulse of infectivity coincides with a maximum in adult female somatic size, which is linked to increased fecundity (Jackson *et al*. 2000). Several studies have identified evidence for increased infestation pressure on farmed salmon related to infective stages from wild salmonids in Ireland (Jackson *et al*. 1997), in association with spring salmon runs in Killary Harbour and grilse runs in Clifden Bay, and in Scotland (Marshall 2003), in association with sea trout in Laxford Bay, Sutherland. Unpublished data from Beartragh Buí Bay, a hydrographically isolated bay where the farm had been fallow for a number of years, shows a sustained level of infestation pressure on autumn smolts stocked in late 2008 through the winter months. The most likely host reservoir to serve as a source of these infestations is resident wild sea trout from the river systems entering into the bay. These river systems support significant populations of sea trout and two of the systems operate as commercial recreational fisheries for sea trout. A sustained level of infestation pressure was recorded from December 2008 through May 2009 with juveniles detected in each month rising to a maximum in May of 4.6 juveniles per fish in the absence of any adult ovigerous females on the farmed fish (D. Jackson, pers. comm.).

In the mid-North Pacific Ocean and Bering Sea, *L. salmonis* was consistently observed on salmon belonging to six *Oncorhynchus* spp. surveyed between 1991 and 1997 (Nagasawa 2001). The parasite occurred on 93.8% of all (*n* = 1267) pink salmon examined, with a mean intensity of 5.9 lice per fish. On chum salmon, the prevalence was 36.4% with a mean intensity of 2.1. Among Chinook, coho and sockeye, *O. nerka* (Walbaum). salmon, and steelhead, *O. mykiss* (Walbaum), trout, the prevalence ranged from 7.8% (sockeye) to 91.5% (steelhead) and the intensity ranged from 1.1 lice (sockeye) to 6.07 lice (steelhead). The consistently high abundance of salmon lice on chum salmon in all years and of pink salmon in alternating years indicates that these species support the vast majority of the *L. salmonis* population in the North Pacific Ocean, supporting earlier work in which pink and chum salmon accounted for 90% of all salmon lice (Nagasawa 1987). The Pacific coast of North America is unique in supporting large populations of anadromous salmonids coincident with the production of Atlantic salmon in seawater netpens (Noakes & Beamish 2011). Surveys conducted along the Pacific coast of North America confirmed the high prevalence of salmon lice observed on the high seas and showed that the proportion of gravid *L. salmonis* ranged from 14% to 36% on chinook, coho, sockeye, pink and chum salmon (Beamish *et al*. 2005). In another study, it was shown that in coastal waters of western Canada and the USA, larger salmon were more heavily infested with *L. salmonis* than smaller salmon (<1 year at sea) (Trudel *et al*. 2007), in agreement with observations by Nagasawa (1987).

### Farmed salmonids

The concentration of infective copepodites is important in the population dynamics of salmon lice. Within an area, the copepodite concentration is dependent on the number of mature females, their fecundity and survival of the nauplii. Water temperature affects female fecundity, development times through all life-cycle stages and probably also survival through all life-cycle stages of salmon lice (Stien *et al*. 2005). This intrinsic temperature dependency is assumed to be a basic driving force for the population dynamics of salmon lice, which has been characterized by annual oscillations in parasite abundance (Lees, Gettinby & Revie 2008b; Jansen *et al*. 2012). Temperature probably affects all life processes in the multi-stage life cycle of salmon lice, but the relationship between temperature and salmon louse population dynamics is not simple. Annual peaks and troughs in the abundance of mobile salmon lice may appear delayed compared with maximum and minimum annual temperature (Jansen *et al*. 2012). Additionally, factors other than temperature may promote cyclic population dynamics. In Pacific Canada, for example, the number of salmon lice peaked in spring and fell to the minimum in late summer. The increase in louse abundance during the autumn and winter periods was suggested to be associated with the spread of infection from wild salmon returning to spawn (Marty, Saksida & Quinn 2010). Factors such as the abundance of plankton-consuming organisms and of pelagic fish serving as unsuitable targets for the copepodites could also influence the survival of the planktonic stages of salmon lice.

Experimentally, salmon louse survival is compromised at salinity levels below 29 parts per thousand (ppt) (Bricknell *et al*. 2006), and nauplii do not develop into infective copepodites at salinities below 25 ppt (Johnson & Albright 1991). Assessing the effects of varying salinity levels on salmon louse infections, however, is complicated by the fact that layers of water with varying salinity levels tend to stratify in the water column, with low-salinity layers on top. Hence, salmon lice may actively avoid low-salinity waters by vertical movement (Heuch 1995). Nevertheless, farms that are exposed to freshwater runoff from rivers are negatively associated with salmon lice abundance (Heuch *et al*. 2009).

Salmon louse nauplii and pre-infection copepodites disperse as plankton in the water currents. Hence, it is expected that water current characteristics will influence salmon louse infections. Hydrodynamic modelling as a basis for tracking salmon louse particles in water currents over time is increasingly being used to study the spread of salmon louse infection (Amundrud & Murray 2009). Such models are in need of broader validation through analyses of salmon louse data. The integration of hydrodynamic modelling and population modelling of salmon lice is a growing field of research where future advances may be expected.

Fish size is associated with salmon louse abundance in farmed fish, such that large fish carry high intensities of infection (Lees *et al*. 2008b; Heuch *et al*. 2009; Jansen *et al*. 2012). This phenomenon can be due to an increased contact rate between infective parasites and large hosts (Tucker, Sommerville & Wootten 2002), and/or the fact that the increased exposure time of large hosts leads to accumulated intensities of infection (Jackson & Minchin 1992). Regardless of the mechanism, the tendency for large salmonid hosts to carry many salmon lice implies that high concentrations of large-sized farmed salmon potentially support large parasite populations. This may be a concern for pest management strategies that involve synchronized production in some areas, that is, towards the late part of the production cycle when all farmed fish within an area have reached a large size.

A key concept in theoretical epidemiology is that increasing host density should promote the population growth of a parasite because the chances of a host contact increase as host density increases, that is, increased transmission increases the parasite reproductive rate (Anderson & May 1991). Salmonid farming in many areas, for example*,* in Norway, results in host densities that massively increase the abundance of salmon lice (Johansen *et al*. 2011), raising the expectation that transmission and population size of the parasite will also increase. This host density effect has been documented at both the level of individual salmon farms and on larger scales. At the farm level, the rate of salmon louse infections on juvenile wild salmon increases during migration past salmon farms, suggesting that salmon louse transmission is associated with farm-produced infectious stages of lice (Krkosek, Lewis & Volpe 2005). Furthermore, a close relationship between estimated numbers of salmon lice on farmed fish and the prevalence of salmon lice on juvenile migrating pink salmon has been demonstrated (Marty *et al*. 2010).

A long-term study (2002–2007) from a Scottish loch suggested that spatial and temporal densities of salmon lice planktonic stages depend on the location of salmon farms (Penston *et al*. 2008). Significant correlations between copepodites in the water column and estimated numbers of gravid female lice on farms were reported, and generally farms with the greatest number of salmon were suggested to contribute more to the densities of copepodites in the water column than farms with fewer fish (Penston & Davies 2009).

In a large-scale study covering all cohorts of farmed salmonids in Norway over the years 2002–2010, local densities of farmed fish were found to affect parasite numbers as well as efforts to control infections. Farms situated in high-density clusters reported generally higher sea louse abundance, increased frequency of chemotherapy treatments and more frequent use of cleaner fish to control infections. Adding to the effect of local densities of farmed fish was a strong temporal correlation with farm-level reports on salmon louse abundance (Jansen *et al*. 2012). This latter effect is due in part to the close proximity of a high number of susceptible hosts leading to auto-infections.

These studies convincingly demonstrate that the increased host density associated with salmon farming promotes transmission and population growth of the salmon louse. The implication of the host density effect is that management should aim to focus on salmon louse infection pressure, that is*,* accounting for host and parasite densities (Penston & Davies 2009; Jansen *et al*. 2012). These data also suggest that effective countermeasures to sea louse infections must take into consideration host and farm densities in the context of local oceanographic and other environmental conditions.

#### Interactions between farmed and wild salmonids

Salmon farms no doubt have profound effects on the local abundance of some parasites (Bakke & Harris 1998), but the quantitative impact of their effect on wild population sizes of salmon and trout remains controversial. A 99% collapse in the pink salmon population and population extinction in only 3.9 generations were predicted due to the impact of salmon lice originating from Atlantic salmon farms in the Broughton Archipelago, BC, Canada (Krkosek *et al*. 2007). These authors estimated an 80% (range 16–97%) louse-induced mortality for pink salmon juveniles. Nevertheless, the population of pink salmon has steadily increased over the last few years (Brooks & Jones 2008), and the latter authors suggested misinterpretation of data was the basis for the prediction of extinction. The number of pink salmon returning to spawn in the fall predicts the number of female salmon lice on farm fish in the following spring. This accounts for 98% of the annual variability in the prevalence of salmon lice on out-migrating wild juvenile salmon (Marty *et al*. 2010). The latter study concluded that productivity of wild salmon is not negatively associated with either farm louse number or farm fish production, although this conclusion is not without controversy (Krkosek *et al*. 2011).

Nominal catches of wild Atlantic salmon in the North Atlantic (NASCO 2011) have declined since the turn of the twentieth century (Hesthagen & Hansen 1991), indicating a corresponding reduction in stock size. While this says nothing about the quantitative impact of salmon lice on populations or individual stocks, it indicates that non-aquaculture factors contribute to the overall variation in seawater survival of the wild salmon population in the North Atlantic. The size of salmonid stocks in both freshwater and marine environments results from an interaction of many anthropogenic and natural biological and physical factors. The wide array of factors that cause variations in the size of salmon and trout stocks is frequently underestimated or overlooked in attempts to find simple explanations. However, the complexity makes it extremely difficult to separate and quantify the effect on each parameter. Figure 1 shows the relative nominal catch of Atlantic salmon from 1960 until today in countries without or with limited salmon aquaculture (USA, Russia, Iceland, Sweden, Denmark, The Faroe Islands, Greenland, UK [except Scotland], France and Spain), major aquaculture producers of Atlantic salmon in countries with spawning wild Atlantic salmon (Norway, Ireland, Scotland and Atlantic Canada) and Norway as the dominating producer. Nominal catches have significantly declined in all regions, and the pattern of decline is similar among regions. Since 1990, the aquaculture production in Norway has increased 6.5-fold to 1 million tons in 2011, and a recent meta-analysis suggests the decline in population sizes tends to be higher in areas with high density of salmon farms (Otero *et al*. 2011). Despite the decline, the International Council for the Exploration of the Seas (ICES) has estimated that the North-East Atlantic salmon stock complex remains at full reproductive capacity (Committee 2011; ICES 2011).

In Norway, several approaches have been used to investigate infection rates on wild Atlantic salmon and sea trout stocks. Direct measurements of the salmon louse infection rate of wild smolts have been obtained by surface trawling of wild migrating smolts (Holm, Holst & Hansen 2000; Holst *et al*. 2003; Heuch *et al*. 2005). The number of lice on sentinel smolts held in small cages in a fjord during the time of natural smolt migration is also used to estimate infestation rates in addition to catches of sea trout and salmon in gillnets and traps. All methods have limitations, and there are obvious risks for getting skewness in the data (Bjørn *et al*. 2011). Only fish that survived the infestation will be caught, behaviour and catchability will depend on infestation rate, fishing gear is size- and species sensitive, and place and time may not be representative for the overall situation.

Early seawater mortality and performance have also been estimated by treating smolts with anti-lice drugs prior to their release and comparing their recapture rates as adults with untreated control groups. The efficacy of the drugs is limited to weeks or in some cases months (Stone *et al*. 2000a; Hvidsten *et al*. 2007; Skilbrei *et al*. 2008), and it is assumed that differences in ocean survival are caused by infestation of control fish with salmon lice during the early stage of smolt migration. The drug emamectin benzoate is usually administered to the fish, either orally (Slice®) (Skilbrei & Wennevik 2006; Jackson *et al*. 2011a,b; Gargan *et al*. 2012; Skilbrei *et al*. 2012) or by intraperitoneal injection to increase the mean dosage and reduce the variability between individuals (Glover *et al*. 2010; Skilbrei *et al*. 2012). Substance EX (Pharmaq) has also been used (Hvidsten *et al*. 2007; Skilbrei *et al*. 2012). Following the release of hatchery-reared smolts of the Orkla River stock in Trondheimsfjord in mid-Norway from 1996 to 1998 (Hvidsten *et al*. 2007), significantly more treated smolts survived in 1998, coincident with a high rate of salmon louse infection in the wild smolts. Hatchery-reared smolts from Dale River stock in western Norway from 1997 to 1999, and from 2001 to 2009, were also released in an area that houses one of the largest concentrations of fish farms in Norway. Significant differences were detected in 3 of the 35 groups released at different times and sites: the only release in 1997, one in 2002 and one in 2007 (Skilbrei & Wennevik 2006; Skilbrei *et al*. 2012), but there were no tendency in the majority of the release groups suggesting differences in survival between the treated and control groups. Overall, the probability of recapturing a treated compared with an untreated smolt was estimated to have an odds ratio of 1.17:1.

Similar treat-and-release studies have been conducted in Ireland. Releases of Burrishoole grilse stock smolt from western Ireland from 2001 to 2008 resulted in a clear trend in which treated fish returned in higher numbers in 9 of 10 years (Jackson *et al*. 2011b), and the differences were statistically significant in 4 of 10. The magnitude of the differences was not large, however, and no differences were observed between the mean returns of treated and untreated groups (analysis of variance *n* = 20). The authors concluded that the level of infestation pressure by salmon lice experienced by the outwardly migrating smolts was not a consistently significant source of additional marine mortality. Similar results were obtained with three other river stocks in 2002 and 2006 (Jackson *et al*. 2011b). In a separate study, releases of smolts over 3 years in two rivers and 2 years in a third river in Ireland from 2004 to 2006 showed a similar treatment effect (Gargan *et al*. 2012). The recapture of treated fish was significantly higher in three of eight releases and also for the combined data. They concluded that treated smolts were, in general, 1.8 times more likely to return and that salmon louse-induced mortality in adult returns in Ireland can be significant. Significant differences were found both where adjacent farms had no adult female burden (one case) and where there were adjacent farms with adult female lice (two cases).

**Figure 2:**
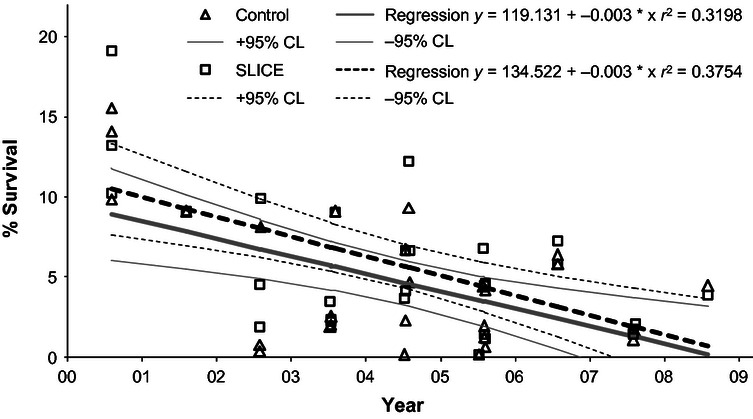
Percentage survival of smolts in Ireland from 2001 to 2009 with 95% confidence limits (CL) fitted. Sea lice infestation was not implicated in the observed decline in survival common to both groups (Jackson *et al*. 2011a). The treated groups were given a prophylactic SLICE treatment giving protection against sea lice infestation for approximately 9 weeks (Jackson *et al*. 2011b).

The results based on these assessments of early sea mortality due to salmon louse infection vary considerably in relation to location, release date and from year to year. The Norwegian investigations, however, indicated a binominal tendency: the numbers of recaptures of treated and control fish were either clearly equal or significantly different. This suggests that salmon lice nauplii are not uniformly distributed in the sea, but rather have a patchy distribution in space and time. This type of variability represents one of the methodological challenges when using release/recapture experiments to produce estimates of early sea mortality due to salmon lice on migrating smolts, especially if there is only one release per location per year. Other potential biases are the large annual variability in ocean survival resulting in low recaptures (Jackson *et al*. 2011b; Skilbrei *et al*. 2012) and uncertainties regarding duration of efficacy against salmon lice afforded by the treatment (Skilbrei *et al*. 2008, 2009; Glover *et al*. 2010). The latter point recognizes the absence of efficacy of emamectin benzoate against salmon lice in several regions. At present, the available information suggests that the majority of the released smolt groups were not at all or only moderately affected by salmon lice, but that some groups were clearly affected. There are also clear local and year-to-year differences in the risk of being too heavily infested with salmon lice. A more up-to-date study, comprising data already published by Jackson *et al*. (2011a,b), together with results published by Gargan *et al*. (2012) and previously unpublished data, including a second time series from a catchment on Ireland's west coast (Jackson, *et al*., 2012), has confirmed these findings. The data, comprising over 350 000 fish from eight locations across nine release dates, show a similar trend in survival between treated and control groups over time when fitted to regression lines (Fig. 2). Analysis based on modelling the percentage return as a binomial response variable, adjusted for location and release-year effects, estimates the probability of returning as 1.14:1 in favour of the treated group, or an absolute difference in sea water returns of approximately 1%. Analysis based on modelling the percentage as a continuous response variable indicates that location (i.e. river; *P* < 0.001) and release date (*P =* 0.001) were both more highly significant than treatment (*P* = 0.034) and an approximately 1% difference between treated and control groups. Thus, estimates from Ireland and Norway indicate an odds ratio of 1.1:1-1.2:1 for sea lice treated Atlantic salmon smolt to survive sea migration compared to untreated smolts. According to the opinion of a Norwegian expert group (Taranger *et al*. 2012), an estimated salmon lice-induced mortality above 10% is expected to have a moderate population regulatory impact, whereas higher mortalities, close to 50%, would have a far greater impact on the affected salmon population.

#### Control: farm monitoring and management thresholds

**Figure 3:**
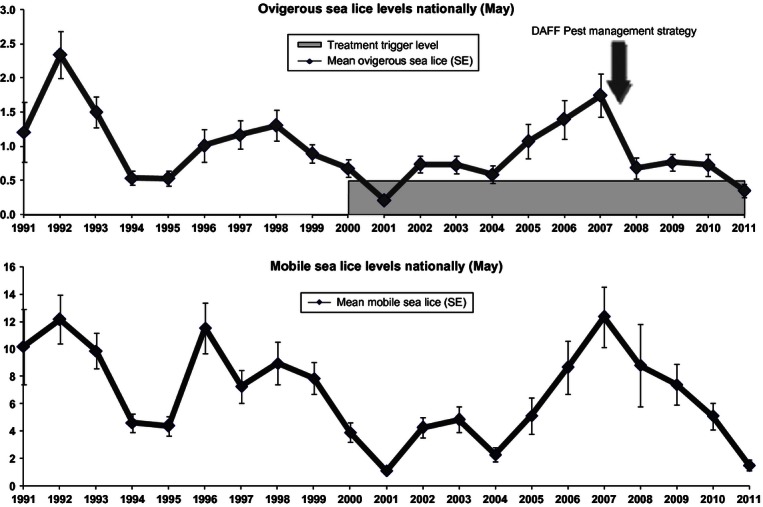
Annual trend (May mean) (SE) of *Lepeophtheirus salmonis* on one-sea-winter salmon.

Lice levels on farmed fish have been monitored in Ireland since 1991, and a comprehensive monitoring programme has been in place since 1993 (O'Donohoe *et al*. 2011). Lice levels on farmed salmon increase with time at sea (Jackson *et al*. 2000) with two sea-winter fish carrying the heaviest burden. Fallowing and separation of generations of farmed fish reduce the effect of sea age on the lice burden (Jackson *et al*. 1997; O'Donohoe *et al*. 2011) of farmed fish. Studies of wild salmon at sea reveal a similar increased abundance of lice with sea age in salmon obtained from north of the Faroe Islands (Jacobsen & Gaard 1997). Salmon lice monitoring and control measures were modified in 2000 and formed the basis of an integrated management protocol for salmon lice in farmed salmon in Ireland (Jackson, Hassett & Copley 2002). Crucial elements of this strategy were identified as separation of generations, annual fallowing of sites and strategic applications of treatments, good fish health management and close cooperation between farms. The monitoring and inspection programme results revealed the benefits of this approach on levels of control achieved from 2000 through 2004 (Fig. 3). From 2005 to 2007, there was a progressive increase in the mean levels of infestation of farmed fish. The increased infestation was identified as resulting from a range of factors, including changes in production practices (Jackson 2011). To address these issues, the Irish authorities issued new guidelines as a Strategy for Improved Pest Control on Irish Salmon Farms in 2008. These guidelines were implemented over the succeeding 2 years and have led to a progressive reduction in the mean levels of infestation (Fig. 3).

In British Columbia, Canada, the autumn rise in *L. salmonis* counts in farmed Atlantic salmon is a consequence of transmission from the large population of returning wild Pacific salmon (Saksida *et al*. 2007a,b). Despite this annual occurrence, routine monitoring of louse numbers on farmed salmon in British Columbia began only in 2003, in response to concerns that infections on juvenile pink salmon were the result of transmission from farmed salmon (Morton *et al*. 2004). A management plan, established in 2003, defined seasonally adjusted salmon louse monitoring and reporting of data and the establishment of action thresholds (Saksida *et al*. 2007a). Since 2007, coordinated treatment with emamectin benzoate applied to farmed salmon 1 or 2 months prior to the migration of juvenile pink and chum salmon into the ocean in the Broughton Archipelago has coincided with a significant and persistent decline in *L. salmonis* abundance on the wild salmon (Jones & Hargreaves 2009; Jones & Beamish 2011). Furthermore, in British Columbia, there is no evidence that the efficacy of emamectin benzoate against *L. salmonis* has changed between 2003 and 2008 (Saksida, Morrison & Revie 2010). In this region, there are ongoing efforts to adequately define salmon lice interactions between wild and farmed salmon using mathematical models (Marty *et al*. 2010; Krkosek *et al*. 2011).

The Salmon Lice Directive (FOR-2009-08-18-1095 2009) provides a framework for the Norwegian surveillance programme for salmon lice in farms. This directive requires that each farm has a general plan for prevention and treatment of salmon lice which as a minimum should contain plans for counting lice in the farm, routines and methods for treatment, including coordinated treatment within the region and documentation of ability to complete treatments within deadlines, routines and methods for evaluation of effect of treatment, routines for use of cleaner fish, routines for fallowing, which other farms are included in the coordinated treatment plan and how the farm seeks to protect wild salmon and trout from negative impacts. An annual update of the plan is required by the Norwegian Food Safety Authority.

This directive imposes a requirement for recording and reporting of data on water temperature, salinity, date and cages counted (minimum 50% of all cages, all if farm has <3), number of sessile lice, number of mobile lice, number of mature females, dates for treatment, drugs used in treatments and possible drug resistance. The key data are published on a weekly basis on http://www.lusedata.no.

Action levels in Norway are 0.5 mature female or three mobile lice on average during the period 1 January through 31 August and one mature female or five mobile during the rest of the year.

The Norwegian Food Safety Authority also has the authority to impose specific and stricter regulations in exposed areas, including reduction in biomass and coordinated treatment and fallowing (FOR-2009-08-18-1095 2009).

#### Control: chemotherapeutants interventions

Over the years, a variety of treatments have been tried against salmon louse infestations. Initially, formaldehyde baths were tested, but proved to have questionable effects (Johannessen 1974). Organophosphates were then introduced, the first being metrifonate as an oral treatment (Brandal & Egidius 1977). However, the low safety margin of oral delivery in salmon led to the introduction of bath applications (Brandal & Egidius 1979). In Scotland, dichlorvos, a related organophosphate, was introduced in 1979 (Rae 1979) and subsequently became the treatment of choice in most salmon-producing countries until the early 1990s when resistance problems became evident (Jones, Sommerville & Wootten 1992; Denholm *et al*. 2002). Alternative bath treatments were launched: first, natural pyrethrins, which were administered as a top dressing in oil at the surface of the pen (Jakobsen & Holm 1990). The administration method, however, was rather impractical. Despite a narrow safety margin, bath treatments with hydrogen peroxide were later introduced, especially in areas where lice had increased tolerance to dichlorvos (Thomassen 1993). In the late 1980s, oral treatments with other compounds were also tried, the first being the macrocyclic lactone ivermectin, which demonstrated a good and long-lasting effect, but also a low margin of safety (Palmer *et al*. 1987). The chitin synthesis inhibitor diflubenzuron was tested in the early 1990s (Horsberg & Hoy 1991); later, another similar compound, teflubenzuron (Branson, Ronsberg & Ritchie 2000), appeared. These compounds had a very broad safety margin, but only targeted the early developmental stages and not adult parasites. In the mid-1990s, azamethiphos, an organophosphate posing less of an occupational hazard than dichlorvos, was launched (Roth *et al*. 1996), after which the synthetic pyrethroids cypermethrin (Hart *et al*. 1997) and deltamethrin (Roth 2000) were introduced. The pyrethroids had a reasonably good safety margin and a good effect on all developmental stages. Finally, in 1999, the macrocyclic lactone emamectin benzoate came to the market (Stone *et al*. 2000b). The safety margin for this orally delivered compound was substantially better than that for ivermectin, and the compound was effective against all developmental stages, lasting up to 10 weeks. After 1999, no new therapeutic agents against salmon lice have been launched. In Norway, the utilization of the different products has been recorded since the early 1980s, and Table 1 illustrates their rise and fall over time.

**Table 1:** Consumption (kilograms active substance) of different agents against salmon lice in Norway from 1981 to 2011 (Grave, Engelstad & Søli 1991; Grave *et al*. 2004; The Norwegian Institute of Public Health 2012)

	Metrifonate (Neguvon)	Dichlorvos (Nuvan)	Azamethiphos (Salmosan)	Hydrogen peroxide	Diflubenzuron (Lepsidon, Releeze)	Teflubenzuron (Ektobann)	Pyrethrins (Py-Sal)	Cypermethrin (Excis, Betamax)	Deltamethrin (Alpha max)	Emamectin (Slice)
1981	4920									
1982	6300									
1983	9810									
1984	14 820									
1985	28 260									
1986	24 860	250								
1987	7390	1310								
1988	3190	3200								
1989	3300	3488								
1990	2408	3416								
1991	2144	3588								
1992	1946	3115								
1993	1779	2470		710 000						
1994	1227	1147	389	290 000			32			
1995		395	738	340 000			26			
1996		161	606	160 000	160	610	9	23		
1997		36	315	20 000	361	1510	18	28		
1998			182		437	1334		3	19	
1999			14		50	231		19	11	4
2000					12	62		73	23	30
2001						28		69	19	12
2002								62	23	20
2003								59	16	23
2004								55	17	32
2005								45	16	39
2006								49	23	60
2007								30	29	73
2008			66					32	39	81
2009			1884	308 000	1413	2028		88	62	41
2010			3346	3 071 000	1839	1080		107	61	22
2011			2437	3 144 000	704	26		48	54	105

Most anti-salmon lice agents act by disrupting neuronal signalling (Roberts & Hutson 1999). Organophosphates inhibit the enzyme acetylcholine esterase, which is responsible for catalysing the hydrolysis of the neurotransmitter acetylcholine at the post-synaptic membrane. Failure to degrade acetylcholine to choline and acetic acid results in continuous neuronal firing, subsequently followed by paralysis and death. The effect is best on pre-adult and adult parasites. Organophosphates (azamethiphos) have a rapid effect that can be recorded after a few hours. The pyrethroids (cypermethrin and deltamethrin) interfere with nerve impulses by modulating the opening and closing of voltage-gated sodium channels in axons, leading to repetitive synaptic discharge, followed by paralysis and death. Pyrethroids are effective against all developmental stages, but the full effect can only be determined after 1–2 weeks, depending on the temperature. Avermectins (emamectin benzoate) modulate specific glutamate- and gamma-aminobutyric acid-gated anion channels. The influx of chloride ions results in hyperpolarization, leading to disruption of nerve impulses, paralysis and death. They are effective against all developmental stages, but the full effect can only be determined after 2–3 weeks. In arthropods, they act through ingestion as stomach poisons, and emamectin benzoate has therefore been developed as a premix for medicated feed. Chitin biosynthesis inhibitors (diflubenzuron and teflubenzuron) are also used as in-feed compounds. They do not interfere with neuronal signalling, instead they inhibit key processes in the chitin synthesis of the parasite, which results in a thin and fragile exoskeleton after moulting. Hydrogen peroxide is a potent oxidizing compound that disrupts membranes. The effect is rapid and most efficacious on pre-adult and adult parasites.

The development of resistance in a parasite population renders an antiparasitic treatment ineffective as was evident in several regions in Norway, where organophosphates in the early and mid-1990s totally lost their effect against salmon lice (Denholm *et al*. 2002). Later, evidence of treatment failures with pyrethroids was reported in Norway, Scotland and Ireland (Sevatdal & Horsberg 2003; Sevatdal *et al*. 2005a).

Treatment failures have also been reported for emamectin benzoate. Initially, these incidents were isolated cases and could frequently be attributed to erroneous calculations of biomass, concurrent diseases and other factors. Appetite varies considerably between individual fish, causing huge variations in the obtained tissue concentrations of orally administered agents (Berg & Horsberg 2009), and this can be misinterpreted as resistance. In 2006, however, several reports from Chile indicated a systematic failure of efficacy by emamectin benzoate towards *Caligus rogercresseyi* (Bravo, Sevatdal & Horsberg 2008). Bioassay tests were established, and the parasites demonstrated significantly reduced sensitivity. In addition, there were reports of reduced efficacy of emamectin against *L. salmonis* in Ireland and Scotland. A Scottish epidemiological survey of salmon lice burdens linked to emamectin treatments between 2002 and 2006 demonstrated a trend towards gradually reduced efficacy. Although salmon lice infestations were reduced following treatments, not all treatments were effective (Lees *et al*. 2008a). In Norway, no comprehensive data have been published, but a survey demonstrated reduced sensitivity towards emamectin benzoate in more than 50% of the salmon lice strains examined (Horsberg 2012). Reduced sensitivity to emamectin benzoate has so far not been recorded on the Pacific coast of Canada (Saksida *et al*. 2011).

Resistance is documented and quantified through efficacy monitoring (parasite counts before and after treatments) and bioassays (toxicological tests of the susceptibility of parasites towards increasing concentrations of the agent in question). Bioassays are labour-intensive and require 50–100 pre-adult parasites per agent (Sevatdal & Horsberg 2003; Sevatdal *et al*. 2005a; Westcott *et al*. 2008). A simplified version, however, is currently under development (Helgesen & Horsberg 2012). Rapid, high-throughput *in vitro* methods, for example*,* quantitative polymerase chain reaction-based assays, would be preferable but are dependent on knowledge about the specific resistance mechanisms. These have only partly been elucidated in salmon lice and include target site alterations (Fallang *et al*. 2004, 2005), enhanced metabolism (Sevatdal *et al*. 2005b) and possibly enhanced elimination mediated by P-glycoprotein efflux pumps (Tribble, Burka & Kibenge 2007; Heumann *et al*. 2012). As several different mechanisms may cause resistance problems, a panel of *in vitro* tests is needed.

The development of resistance in salmon lice, especially *L. salmonis,* is a serious situation. In Norway, Scotland, Ireland and eastern Canada, the number of farmed salmon is far greater than the number of wild salmon. Thus, the main source for re-infestation comes from the farms themselves where regular parasite treatments put a constant selection pressure on resistance development. In these countries, the influx of naive parasites from wild fish hosts is limited. Thus, the problem will not disappear by itself. New chemicals may only be valuable for a limited time period. Management practices with a variety of non-chemical control methods, preservation of sensitive parasites, coordinated production zones, synchronized treatments and synchronized fallowing of sites in larger areas seem to be the most promising strategy to handle the problem.

#### Control: cleaner fish

The use of wrass as cleaner fish for salmon lice control was developed in the late 1980s (Bjørdal 1988a,b, 1990). The aquaculture industry has depended on wild catches for their supply, and several species are used, for example, goldsinny wrasse, *Ctenolabrus rupestris*, ballan wrasse, *Labrus bergylta* Ascanius, corkwing wrasse, *Symphodus melops* (L.), rock cook, *Centrolabrus exoleus* (L.), cuckoo wrasse, *Labrus bimaculatus* L., and scale-rayed wrasse, *Acantholabrus palloni* (Risso) (Espeland *et al*. 2010). Goldsinny wrasse, ballan wrasse and corkwing wrasse are the species used most frequently in Norway (Blom 2010). Stocking density of approximately 4 wrass per 100 salmon is common, slightly more for small wrasses as goldsinny and less for larger wrasses. Ballan wrasse are efficient and are often used at rates of 1 per 100 salmon. The successful use of cleaner fish depends on healthy fish and clean cages. Wrass require shelter for well-being and readily seek alternative feed sources if the nets are overgrown. Recent experiments have shown that lumpfish, *Cyclopterus lumpus* L., can be used as cleaner fish and that their aquaculture production is possible (Anon 2012).

The ballan wrasse is the largest of the European wrass, attaining a maximum size of approximately 60 cm (Quignard & Pras 1986), and is therefore a suitable size for being kept in cages with large salmon (3–6 kg) (Muncaster 2008). Based on their wide geographical distribution, they tolerate a wide range of environmental conditions and have been shown to survive in cages over winter in Norway (Bjelland *et al*. 1996). Over the last few years, there has been a growing interest in farming wrass, with a focus on the ballan wrasse (I. Opstad, P.G. Kvenseth, P. Jensen and A.B. Skiftesvik, unpubl. data). Although weaning the ballan wrasse from live food to a formulated diet is challenging, I. Opstad, P.G. Kvenseth, P. Jensen and A.B. Skiftesvik (unpubl. data) developed a successful weaning diet that supports survival up to 88%.

**Figure 4:**
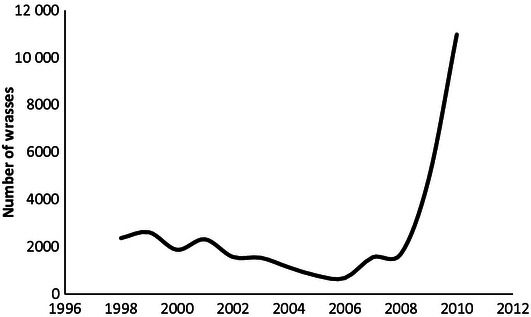
Catches of wrass in Norway for use as cleaner fish in salmon cages (Fiskeridirektoratet 2012).

The rapid increase in wrasse fisheries has raised concerns (Fig. 4), as their biology, ecology and population dynamics are poorly known. Many species change sex during their lifetime, and some have longevity of 20–25 years and are territorial. These species may therefore be vulnerable to overexploitation (Espeland *et al*. 2010).

#### Control: vaccination

There are two published studies in which vaccine candidate antigens were tested against salmon lice. Grayson *et al*. (1995) extracted proteins from adult *L. salmonis* and used these antigens to immunize Atlantic salmon. They found a significant reduction in gravid female lice on the vaccinated fish, and the lice also produced fewer eggs. More recently, Carpio *et al*. (2011) made a recombinant antigen from the novel my32 gene obtained from *C. rogercresseyi*. The my32 from *C. rogercresseyi* is similar to a protective antigen from ticks (Almazan *et al*. 2005). Carpio *et al*. (2011) found that immunization with recombinant my32 resulted in a significant reduction in the number of *C. rogercresseyi* 24 h post-infection and a delayed developmental rate of immunised fish. Further analysis of the results showed that the vaccine effect was due to the reduced settlement of larvae produced by lice on the immunized fish. The overall effect of the vaccine in that experiment was 57% inhibition of infestation. A homologue to the my32 antigen from *C. rogercresseyi* is also present in *L. salmonis*. Using this as a vaccine antigen in Atlantic salmon, however, did not significantly reduce the number of lice or lice fitness (F. Nilsen, R. Skern-Mauritzen, C. Eichner and S. Dalvin, unpubl. data). Despite these differences, studies of *C. rogercresseyi* point to the possibility of a future commercial salmon lice vaccine.

Recent developments in sequencing technology have led to a decrease in the sequencing costs and large increase in sequencing throughput. The salmon louse genome has been sequenced and assembled, and final annotation is in progress (Skern-Mauritzen *et al*. 2012). This means that all potential treatment targets will be available, and an approach utilizing all this information is possible. A preliminary count indicates about 22 000 genes occur in the salmon louse, although only a small fraction of these would be useful as vaccine antigens. A key challenge is therefore to identify the best candidates that could be used in a future commercial vaccine. Recently, Maritz-Olivier, Van Zyl & Stutzer (2012) proposed a functional genomics approach to identify vaccine candidates in cattle ticks. Their approach is based on several steps of *in silico* evaluation of candidates prior to experimental verification of antigenicity and testing of recombinant antigens in trial vaccines. Although the current knowledge of efficient protective antigens against ectoparasites is limited, using all the genomic information in the initial analysis will be very helpful towards identifying a number of vaccine antigen candidates. During the last years, a set of tools and resources were established that facilitate the development of new control tools for salmon lice. Efficient and accurate experimental facilities are crucial for the development of new treatment methods for salmon lice. Hamre, Glover & Nilsen (2009) established specific laboratory strains of *L. salmonis* along with procedures for maintaining and breeding salmon lice. A refined and more accurate set-up for conducting experiments with salmon lice was also recently developed (Hamre & Nilsen 2011). In addition, a set of molecular methods was established that will facilitate research leading to new treatment methods. Examples are lice-specific microarrays (Eichner *et al*. 2008; Sutherland *et al*. 2012) and systemic RNA interference methods (Dalvin *et al*. 2009), which will be very useful in the future.

Classical bacterial and virus vaccines enhance the resistance to infection by limiting the capacity for pathogen replication within the host. For parasites like ticks and salmon lice that do not proliferate on or in the host, the situation is quite different, and for these parasites, a vaccine that reduces the number of offspring has a direct effect on new infections. For the commercially available cattle tick vaccine, the overall effect of the vaccine is about a 90% reduction in the tick reproduction capacity. This includes increased tick mortality and a reduction in the number of eggs produced per female. The obvious question is whether a vaccine effect similar to that observed for cattle ticks is sufficient to make a difference for salmon lice. For example, a salmon louse vaccine that reduces the number of eggs/female by 50% will be comparable with a 50% reduction in the number of female lice/fish. A vaccine like the cattle tick vaccine or a vaccine against salmon lice with a similar level of effect will not be a stand-alone tool in parasite control. Together with other antiparasitic measures, however, the effect will be large. The reliance on chemotherapeutants will be reduced, and the lifetime for valuable medicine will be extended. If all farmed fish are vaccinated, the vaccine would also be effective on escapees and hence contribute further to lice control.

## Conclusions

Salmon lice are natural parasites on salmonids in the sea water with a circumpolar distribution in the northern Hemisphere. The populations in the Atlantic and Pacific oceans are genetically distinct. Intensive salmon farming has improved the conditions for the growth and transmission of the parasites compared with natural conditions. Gene flow among populations appears high and most likely results from association with highly migratory hosts. There are distinct differences in the susceptibility to salmon lice infections among salmonid fish species.

Salmon recreational fishery, commercial fishery (sea fishery) and aquaculture have different stakeholders, practices, traditions and management objectives and strategies (Liu, Olaussen & Skonhoft 2011). Sea lice have clearly impacted wild salmon and trout fisheries without compensating for the imposed negative external costs. The quantitative estimates of these impacts show large variations. Further research is needed in order to understand the mechanisms and processes. The density of farms in an area has a clear effect on the levels of sea lice at individual farms within that area.

Since the start of large-scale salmon farming in the 1970s, control of salmon lice has been based mainly on chemotherapy. This has been effective and simple to use, but also creates unwanted environmental effects, occupational hazards and drug resistance problems. During the last few years, there has been a trend towards a more integrated management approach with synchronized treatments, biological control (cleaner fish), immunological interference (immunostimulants), mechanical de-lousing systems, selective breeding for louse-resistant salmon and regulatory approaches (zones with synchronized production and fallowing).

Sea lice resistance to chemotherapeutants is a serious concern. In Norway, Scotland, Ireland and eastern Canada, the number of salmon in farms greatly exceeds the number of wild salmon. Thus, the main sources of re-infestation are the farms themselves, where regular parasite treatments place constant selection pressure on resistance development. New chemicals may only be valuable for a limited period of time. Management practices with a variety of methods will be necessary to keep the sea lice under control in salmon farms.

Two published studies tested vaccine candidate antigens against salmon lice, which resulted in a reduced infection rate (Grayson *et al*. 1995; Carpio *et al*. 2011). For parasites like salmon lice that do not proliferate on or in the host, a vaccine will primarily reduce infection pressure. Salmon lice create problems for both the salmon farming industry and, under certain conditions, wild salmonids. A vaccine will probably not be adequate as a stand-alone treatment, but it would be a valuable element in the hierarchy of salmon lice prevention methods.

For the foreseeable future, salmon lice will continue to be a serious problem for the salmon farming industry and a threat to their environmental credibility. Salmon farmers invest in expensive sea lice monitoring and treatment programmes. The key to a sustainable production is to integrate several management practices. This will require a substantial increase in research in areas such as new pharmaceuticals, mechanical lice removal, vaccines and immunostimulants, selective breeding for increased resistance, effective aquaculture production and use of cleaner fish, and the development of coastal hydrographic models to estimate transmission dynamics and to support farm siting decisions and coordinated management.
